# Elusive physiological role of prostatic acid phosphatase (PAP): generation of choline for sperm motility via auto-and paracrine cholinergic signaling

**DOI:** 10.3389/fphys.2023.1327769

**Published:** 2023-12-22

**Authors:** Peter J. Hanley

**Affiliations:** IMM Institute for Molecular Medicine, HMU Health and Medical University Potsdam, Potsdam, Germany

**Keywords:** prostatic acid phosphatase (PAP), phosphocholine (PC), choline, cholinergic signaling, sperm motility, acetylcholine (ACh), acetylcholine receptor (AChR)

## Abstract

Prostatic acid phosphatase (PAP) exists as two splice variants, secreted PAP and transmembrane PAP, the latter of which is implicated in antinociceptive signaling in dorsal root ganglia. However, PAP is predominantly expressed in the prostate gland and the physiological role of seminal PAP, first identified in 1938, is largely unknown. Here, the author proposes that PAP, following ejaculation, functions to hydrolyze phosphocholine (PC) in seminal fluid and generate choline, which is imported by sperm via a choline transporter and converted to acetylcholine (ACh) by choline acetyltransferase. Auto- and paracrine cholinergic signaling, or choline directly, may subsequently stimulate sperm motility via α7 nicotinic ACh receptors (nAChRs) and contractility of the female reproductive tract through muscarinic ACh receptors (mAChRs). Consistent with a role of PAP in cholinergic signaling, 1) seminal vesicles secrete PC, 2) the prostate gland secretes PAP, 3) PAP specifically catalyzes the hydrolysis of PC into inorganic phosphate and choline, 4) seminal choline levels increase post-ejaculation, 5) pharmacological inhibition of choline acetyltransferase inhibits sperm motility, 6) inhibition or genetic deletion of α7 nAChRs impairs sperm motility, and 7) mAChRs are expressed in the uterus and oviduct (fallopian tube). Notably, PAP does not degrade glycerophosphocholine (GPC), the predominant choline source in the semen of rats and other mammals. Instead, uterine GPC phosphodiesterases may liberate choline from seminal GPC. In summary, the author deduces that PAP in humans, and uterine GPC phosphodiesterases in other mammals, function to generate choline for sperm cholinergic signaling, which promotes sperm motility and possibly contractility of the female reproductive tract.

## 1 Introduction

Sperm maturation, including the capacity for motility and fertilization, increases along the length of the epididymis, between caput epididymis and cauda epididymis (Bedford, 1963; Yeung et al., 1993). Following ejaculation, sperm combine with secretions from the accessory sex glands, the prostate and seminal vesicles. These secretions not only promote sperm motility (Yanagimachi, 2022), but also temporarily ensnare the sperm. Zn^2+^-binding semenogelin secreted by the seminal vesicles forms a gel-like matrix (Lilja and Laurell, 1984; Lilja and Laurell, 1985; Lilja et al., 1987; Anamthathmakula and Winuthayanon, 2020), seminal coagulum (copulatory plug), which traps sperm, accounting for only 2%–5% of the seminal fluid volume, and prevents premature capacitation (Juyena and Stelletta, 2012), the ability of sperm to fertilize an oocyte (Chang, 1951; Austin, 1952; Puga Molina et al., 2018; Yanagimachi, 2022). The gel-like clot formed by semenogelin not only helps to retain sperm within the female reproductive tract, but it also provides protection from the hostile acidic environment of the vagina. The prostate secretes Zn^2+^-inhibited prostate-specific antigen (PSA), a serine protease, which becomes activated following Zn^2+^ sequestration by semenogelin (Lilja et al., 1987; Malm et al., 2007; Anamthathmakula and Winuthayanon, 2020). Activated PSA cleaves semenogelin, thereby liquefying the semen within 15–20 min (Malm et al., 2007). Semenogelin, also known as seminal vesicle secretion 2, is encoded by a single gene, *Semg1*, in mice and by two genes, SEMG1 and SEMG2, in humans. Kawano et al. (2014) showed that homozygous *Semg1* knockout male mice produce smaller litter sizes and copulatory plugs are not formed in female mice mated with male homozygous mutants. However, the percentages of motile sperm and hyperactivated sperm, a state of highly vigorous motility (acquired in the oviduct) first described by Yanagimachi (2022), are not decreased in homozygous *Semg1* knockout mice. The authors also showed that uterine fluid, but not fluid from the ampulla of the oviduct, is cytotoxic to sperm, and deduced that semenogelin provides protection from the spermicidal environment of the uterine cavity. Thus, the mixing of fluids from the prostate (Zn^2+^-inhibited PSA) and the seminal vesicles (Zn^2+^-binding semenogelin) enable the formation of a gel and its subsequent liquefaction, which releases sperm with a protective coat. In addition to semenogelin, a thick glyocalyx, acquired during maturation in the epididymis, help sperm to cross the uterus and survive uterine immunity (Tecle and Gagneux, 2015; Lan et al., 2020).

In addition to PSA, the prostate secretes prostatic acid phosphatase (PAP), encoded by ACPP in humans and *Acp3* (acid phosphatase 3) in mouse. The physiological function of this enzyme in seminal plasma is unclear. In this mini-review, the author distills and analyzes the literature, providing an evidence-based framework for understanding the physiological role of seminal PAP, while also shedding new light on the functions of seminal choline and cholinergic signaling in sperm.

## 2 Antinociceptive function of PAP in dorsal root ganglia

PAP exists in two widely expressed isoforms in both human and mouse, generated by alternative splicing that either includes or excludes a C-terminal transmembrane domain: transmembrane PAP, a type-I transmembrane protein, and secreted PAP (Quintero et al., 2007). Both isoforms exhibit 5′-ectonucleotidase activity and accordingly degrade (extracellular) adenosine monophosphate to adenosine (Zylka et al., 2008; Street et al., 2013; Araujo et al., 2014). Transmembrane PAP is the predominant isoform expressed in dorsal root ganglia, where it has been deduced to generate adenosine and exert an antinociceptive effect through stimulation of adenosine A_1_-receptors (Zylka et al., 2008). Nevertheless, in human, PAP is predominantly expressed in the prostate and from hereon the abbreviation PAP refers to the secreted form.

## 3 PAP liberates choline from seminal gland-secreted phosphocholine

**FIGURE 1:**
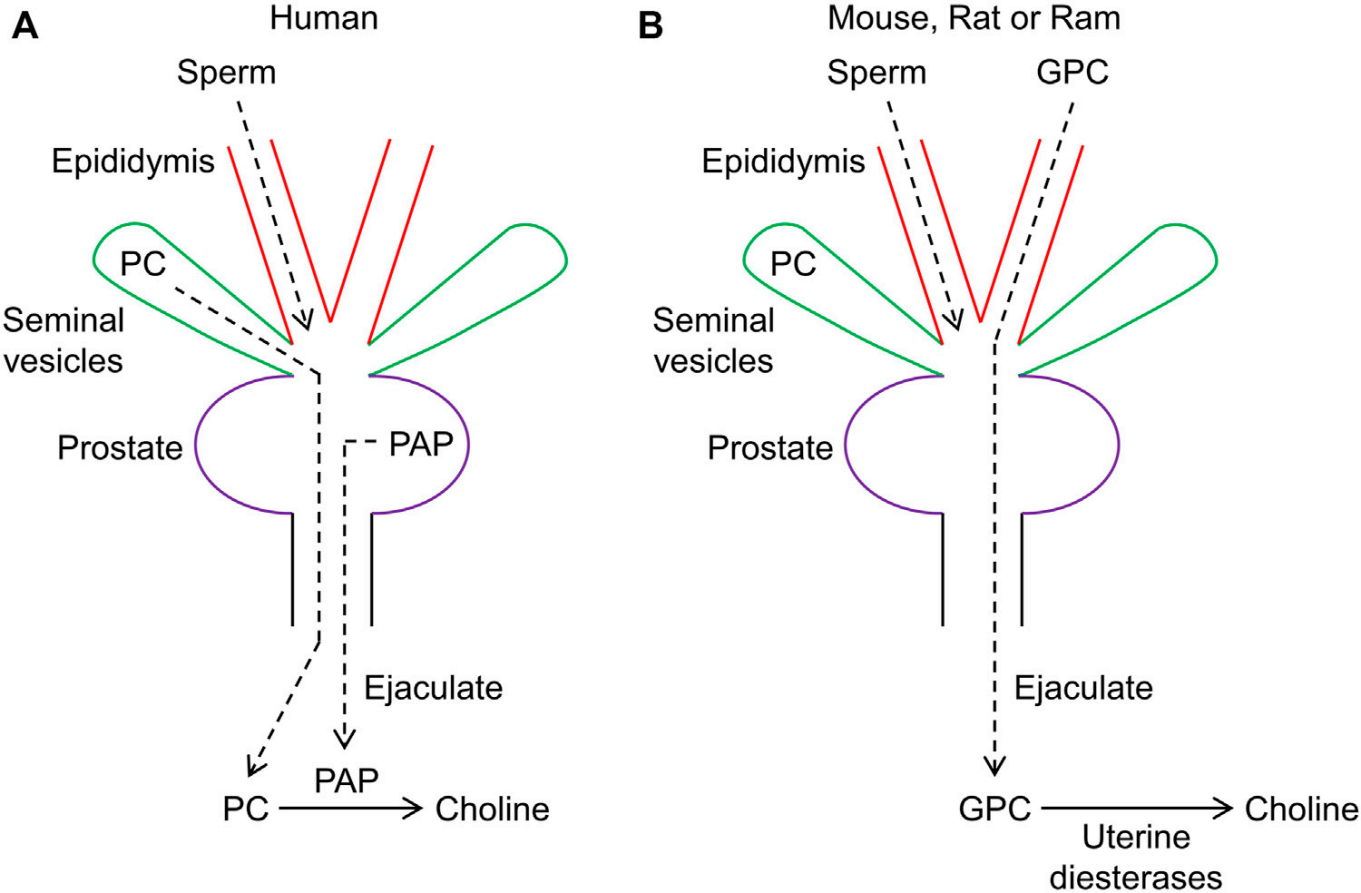
Schematic diagram depicting seminal choline production in humans through prostatic acid phosphatase (PAP) and in other mammals via uterine diesterases. **(A)** in humans, PAP serves to hydrolzye phosphocholine (PC) and produce choline. **(B)** in mice and other mammals, choline is primarily produced through uterine diesterases acting on glycerophosphocholine (GPC), which is primarily secreted by the epididymis.

Lundquist reported in 1946 (Lundquist, 1946) that the levels of inorganic phosphate are low immediately following ejaculation, but rapidly increase within minutes. The author deduced that inorganic phosphate and choline are produced when an acid phosphatase secreted by the prostate, previously described by Kutscher and Wolbergs (1935), hydrolyzes phosphocholine (PC), secreted by the seminal glands. Thus, the mixing of PC from seminal vesicles and PAP from the prostate produces choline in the ejaculate, as schematically shown in Figure 1. Consistent with this model, PC has been shown to be a highly specific substrate for PAP (Seligman et al., 1975; Serrano et al., 1977), a tartrate-sensitive member of the histidine phosphatase superfamily (McTigue and Van Etten, 1978; Jakob et al., 2000; Rigden, 2008; Ara et al., 2013). Indeed, PC is hydrolyzed by human PAP (Seligman et al., 1975; Serrano et al., 1977), but not human liver acid phospatase (Saini and Van Etten, 1978), and this substrate specificity has been exploited in the past to quantify serum phosphatase activity originating from the prostate (Saini and Van Etten, 1981). PAP activity per milligram of prostate varies widely among species and is notably 1,000 times or more higher in humans than rodents (Seligman et al., 1975).

In further work, Lundquist (1953) observed that the mixing of saline extract from the prostate of the rat with extracts from the seminal vesicles produced much more free choline than could be accounted for by hydrolysis of available PC. This suggested the presence of an alternative source of choline, which was identified as glycerophosphocholine (GPC). High levels of GPC, relative to PC and normalized to unit moist tissue weight, were found in rat, guinea-pig, and rabbit in the order: rat > guinea-pig > rabbit. Dawson et al. (1957) confirmed that PC is the predominant source of choline in human seminal plasma. Human semen also contains some GPC, but unlike PC, it does not degrade during semen incubation (Dawson et al., 1957). In contrast to humans, high levels of GPC, but only trace levels or no PC, were detected in the semen of ram, bull, goat, boar, rat, and rabbit. The source of GPC has been localized to the epididymis (Scott et al., 1963; Wallace et al., 1966; Arrata et al., 1978; Hinton and Setchell, 1980; Hoffmann and Killian, 1981) (Figure 1). Assuming that choline plays a role in fertility, the lack of infertility or subinfertility observed in mice lacking *Acp3* (Quintero et al., 2013) could be explained by the availability of an alternative substrate, GPC, for generation of choline. However, *Acp3* knockout mice develop prostate adenocarcinoma (Quintero et al., 2013) and exhibit susceptibility to chronic pain (Zylka et al., 2008). White and Wallace (1961) showed that secretions from the female reproductive tract could hydrolyze GPC. Preheating of uterine secretions to 100 °C for 5 min prevented GPC hydrolysis, suggesting that an enzyme, presumably a diesterase, is responsible. A Ca^2+^-dependent diesterase, with exclusive specificity for GPC, has been purified from rat uterine secretions (Mitra and Chowdhury, 1994). This enzyme catalyzes the hydrolysis of GPC to choline and glycerol 3-phosphate. A likely candidate for this enzyme is ectonucleotide pyrophosphatase/phosphodiesterase (ENPP) family member 6 (ENPP6), which has substrate specificity for GPC (Borza et al., 2022). ENPP6 exists in two forms: a glycosylphosphatidylinositol (GDI)-anchored enzyme and, after cleavage of the GDI-anchor, a soluble enzyme. However, ENPP6 is predominantly expressed in the brain and kidney (Greiner-Tollersrud, 2014), although it has been detected in bovine uterine fluids (Piibor et al., 2023). Glycerophosphodiester phosphodiesterase domain containing 5 (GDPD5) is widely expressed in humans, including in the uterus, and can also generate choline from GPC (Gallazzini et al., 2008; Lang et al., 2008). Deletion of *Gdpd5*, previously denoted *Gde2*, in mice and interbreeding produced homozygotes in the expected Mendelian ratios, which exhibited normal fertility (Sabharwal et al., 2011), although abnormal cortical neuron development was observed (Sabharwal et al., 2011; Rodriguez et al., 2012). Alternatively, choline can also be generated in the uterus by the secreted enzyme ENPP2 (Ahn et al., 2011), which converts lysophosphatidylcholine (LPC) to lysophosphatidic acid (LPA) (Goding et al., 2003; Seo et al., 2012). Mice lacking ENPP2 (encoded by *Enpp2*), also known as autotaxin or lysophospholipase D, die at midgestation and exhibit abnormal vascular development (van Meeteren et al., 2006), whereas homozygous *Enpp6* knockout mice are viable and fertile (Morita et al., 2016). Interestingly, human semen has been shown to exhibit ENPP2 activity and PAP was deduced to degrade the product LPA (Tanaka et al., 2004), thereby driving the ENPP2-catalyzed reaction through mass action to produce more choline. Thus, whereas choline can be produced from PC by PAP in humans, choline can be derived from GPC, or possibly LPC, in other mammals via various uterine phosphodiesterases.

Choline production within the female reproductive tract after sexual intercourse seems to be a shared phenomenon across various species, suggesting that this quaternary ammonium cation plays a specific role in sperm function and fertility. On one hand, choline serves as a precursor for the synthesis of specific phospholipids, including phosphatidylcholine and sphingomyelin. On the other hand, choline is essential for the production of the neurotransmitter acetylcholine (ACh). While the notion of sperm synthesizing ACh may appear counterintuitive, it should be noted that non-neuronal cholinergic (ACh-mediated) signaling has been extensively implicated in the reproductive system (Wessler and Kirkpatrick, 2017).

## 4 Hypothesis: choline derived from PC, GPC, or LPC drives sperm motility, as well as uterine contractility, via auto- and paracrine cholinergic signaling

**FIGURE 2:**
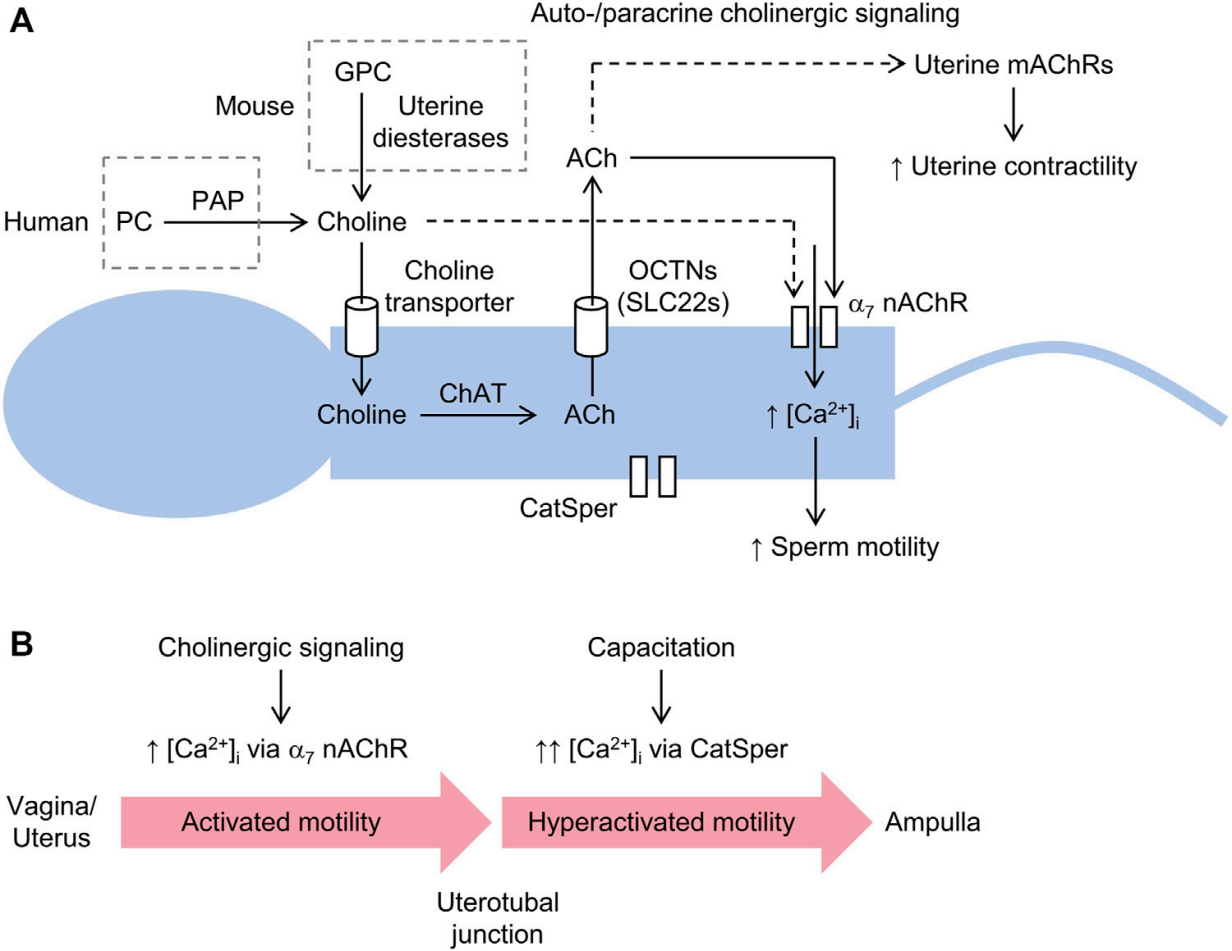
Schematic diagram of sperm motility induced by the generation of choline via prostatic acid phosphatase (PAP) and driven by cholinergic signaling. **(A)** choline liberated from either phosphocholine (PC) in humans or glycerophosphocholine (GPC) in other mammals is imported into sperm via a choline transporter and converted to acetylcholine (ACh) through choline acetyltransferase (ChAT). Sperm are unable to store ACh, which is released to the extracellular space via an organic cation transporter novel (OCTN) or other transporter, where it stimulates Ca^2+^-permeable α7 nicotinic ACh receptors (nAChRs). Increased intracellular [Ca^2+^] ([Ca^2+^]_i_) promotes flagellar beating and sperm motility. Choline can directly activate α7 nAChRs, albeit with less potency than ACh. Sperm also express a Ca^2+^-selective channel, known as the cation channel of sperm (CatSper), which is not required for initial post-ejaculation activated motility, but is essential for hyperactivated motility and fertility. **(B)** cholinergic signaling induced by the availability of free choline (shown above) probably serves to promote so-called activated motility, characterized by low-amplitude and symmetrical beating of the flagellum, and migration of sperm from the vagina and/or uterus to uterotubal junctions. In the oviduct, capacitation leads to the activation of CatSper, which is required for hyperactivated motility, characterized by high-amplitude and asymmetrical beating of the flagellum, and fertility, in particular, penetration of the cumulus oophorus and zona pellucida.

The author speculates that choline is taken up by sperm via a choline transporter and converted via choline acetyltransferase (ChAT) to ACh, which is subsequently released via a presumably non-vesicular pathway to stimulate sperm motility and/or uterine contractile activity via auto- and paracrine activity, respectively, as schematically illustrated in Figure 2A. Pharmacological inhibition of ChAT, which catalyzes the synthesis of ACh by transferring an acetyl group from acetyl-CoA to choline, has been shown to inhibit the motility of human sperm (Sastry et al., 1981). Consistent with these observations, sperm have been shown to express *Chat* mRNA and immunofluorescence imaging revealed intense localization of ChAT to the post-acrosomal region in the head of mature sperm (Ibáñez et al., 1991). Genetic deletion of *Chat* in a mouse model could help to elucidate putative roles of cholinergic signaling in sperm motility and/or fertility. However, ChAT is critical for neuronal cholinergic signaling and deletion of *Chat* in mice is lethal (Misgeld et al., 2002). Homozygous *Chat* knockout mice exhibit flaccid paralysis and die shortly after birth. This lethal phenotype could be explained by loss of presynaptic ACh synthesis since ChAT activity could not be detected in brain lysates from homozygous mutants and stimulation of motor neurons failed to evoke excitatory postsynaptic potentials in myotubes from homozygous mutant embryos. In principle, lethality could be circumvented by crossing floxed *Chat* mice, such as B6; 129-*Chat* ^tm1Jrs^/J mice, in which *loxP* (Cre recombinase-recognition) sites flank exons 3 and 4 of the *Chat* gene, with a sperm-restricted Cre recombinase mice, such as Stra8-Cre mice (Sadate-Ngatchou et al., 2008), in which Cre recombinase expression is driven by the gene *Stra8* (stimulated by retinoic acid gene 8), a gene specifically activated in spermatocytes as they enter meiosis.

In addition to *Chat*, sperm have been shown to express the high-affinity choline transporter SLC5A7, encoded by *Slc5a7* in rodents (Schirmer et al., 2011). Deletion of *Slc5a7* in mice, similar to deletion of *Chat*, is lethal. Homozygous *Slc5a7* knockout mice appear morphologically normal immediately after birth, but exhibit immobility and severely impaired breathing, which leads to cynanosis and death within 1 hour (Ferguson et al., 2004), which can be explained by lack of availability of the neurotransmitter ACh presynaptically at neuromuscular junctions. As in the case of *Chat*, conditional deletion of *Slc5a7* in the sperm of mice could help to confirm whether the choline transporter SLC5A7 is important for sperm motility and/or fertility. Mice harboring the knockout first allele (tm1a), *Slc5a7* ^tm1a(KOMP)Wtsi^ mice, which can be converted to a floxed allele (tm1c), have been produced by the International Mouse Phenotyping Consortium (IMPC) and could be used to generate sperm-restricted *Slc5a7* knockout mice, for example, by crossing floxed *Slc5a7* mice with Stra8-Cre mice.

Acetylcholine synthesized via ChAT within sperm, which lack the capacity for its storage (Bishop et al., 1976), is presumably released through a cation transporter, as shown in Figure 2A. Human sperm express members of the organic cation transporter novel (OCTN) subfamily of the solute carrier (SLC) superfamily, including OCTN1 and OCTN2 (Xuan et al., 2003). OCTN1, encoded by *Slc22a4* in mouse, has been shown to transport ACh, which is competitively inhibited by the synthetic transporter substrate tetraethylammonium (Pochini et al., 2012; Pochini et al., 2019). Mice lacking *Slc22a4* are fertile, but sperm motility was not investigated (Kato et al., 2010). However, ACh, as well as choline and carnitine, may be transported to various degrees by other OCTNs expressed in mouse sperm (Kobayashi et al., 2007). Notably, both human and rat OCTN1 and OCTN2, heterologously expressed in oocytes from *Xenopus laevis*, have been shown to release ACh (Lips et al., 2005). Released ACh could stimulate ACh receptors expressed on sperm or in the female reproductive tract. Indeed, ACh has been shown to increase sperm motility in various species, including sea urchin, bull, chimpanzee, and human (Nelson, 1972; McGrady and Nelson, 1976; Dwivedi and Long, 1989), whereas findings for mouse are inconsistent (Sliwa, 1995; Makino et al., 2021).

ACh receptors are classified into two main types: nicotinic ACh receptors (nAChRs), which are ACh-gated ion channels mainly found in the nervous system and skeletal muscle, and muscarinic ACh receptors (mAChRs), G protein-coupled receptors predominantly located at targets of the parasympathetic nervous system, including the heart, smooth muscle, and glands. nAChRs are heteropentamers permeable to Na^+^ and K^+^ and consist of two identical α-subunits and three distinct subunits, which can be β, γ, δ, or ε (Skok, 2022). Homopentameric α7 nAChRs have also been identified, which are additionally permeable to Ca^2+^ (Delbono et al., 1997). Interestingly, choline has been shown to be a selective full agonist of α7 nAChRs (Papke et al., 1996; Alkondon et al., 1997; Mike et al., 2000), albeit with one order of magnitude lower potency than ACh (Alkondon et al., 1997). Mice lacking *Chrna7*, which encodes the α7 subunit, are viable and exhibit no obvious developmental or neurological disorders (Orr-Urtreger et al., 1997). Homozygous male and female *Chrna7* knockout mice were reported to be fertile, but the authors raised nonspecified potential fertility issues. Indeed, Bray et al. (2005) found that sperm from *Chrna7* knockout mice had significantly impaired motility and a lower rate of hyperactivation, whereas the number, morphology, and viability of sperm were normal. These findings align with the observation that various nAChR inhibitors, including the snake toxin α-bungarotoxin, as well as other nAChR antagonists like hexamethonium and succinylcholine, inhibit sperm motility (Nelson, 1976; Dwivedi and Long, 1989). In addition to the α7 subunit, the inhibitor α-bungarotoxin binds to the α9 subunit, which is also expressed in sperm (Kumar and Meizel, 2005). Immunofluorescence imaging revealed distinct localization patterns of α7 and α9 subunits in sperm. Specifically, α7 subunits are prominently localized to the post-acrosomal regions encompassing the head, neck (connecting piece), midpiece, and the principal and terminal pieces of the tail (flagellum) (Bray et al., 2005; Kumar and Meizel, 2005) In contrast, α9 subunits exhibit a more restricted localization, being detectable in approximately 50% of sperm and primarily localizing to the acrosomal region (Makino et al., 2021). Makino et al. (2021) did not observe increased motility with ACh treatment, but instead the acrosome reaction rate was decreased, suggesting that nAChRs containing α9 subunits regulate the acrosome reaction.

Extracellular Ca^2+^ is essential for sperm motility, highlighted by the observation that removal of Ca^2+^ halts motility, while reintroduction reactivates it (Feng et al., 1988; Aaberg et al., 1989; Ignotz and Suarez, 2005; Jin et al., 2007; Torres-Flores et al., 2011). Auto- and paracrine cholinergic signaling, and possibly direct choline-mediated signaling, may increase intracellular [Ca^2+^] and promote the low-amplitude and symmetrical mode of flagellar beating associated with activated motility (Turner, 2006), required for freshly ejaculated sperm to move with high directionality through the female reproductive tract (Turner, 2006; Suarez, 2016). In addition, non-neuronal cholinergic signaling driven by choline availability may also stimulate peristaltic smooth muscle contractions of the female reproductive tract, which help to propel sperm, or sperm aggregates, towards and beyond the uterotubal junctions (Wang and Larina, 2023). That is, following ejaculation, choline may directly stimulate Ca^2+^ influx into the tail via activation of α7 nAChRs or indirectly via the pathway of choline uptake, ACh synthesis, and ACh release (Figure 2A). Cholinergic receptor stimulation is probably cyclical due to a combination of ACh hydrolysis by acetylcholinesterases (AChEs), expressed in sperm (Nelson, 1964; Chakraborty and Nelson, 1976; Stewart and Forrester, 1978; Egbunike, 1980; Egbunike, 1982), and α7 nAChR desensitization (Papke et al., 2009). At the isthmus of the oviduct, sperm accumulate (Suarez, 2008) and capacitation procedes, involving removal of plasma membrane cholesterol and intracellular alkalinization, which leads to activation of a Ca^2+^-selective channel, known as the cation channel of sperm (CatSper) (Ren et al., 2001; Kirichok et al., 2006). This channel is localized to the principal piece of sperm (Chung et al., 2017; Wang et al., 2021) and is essential for hyperactivated motility (Turner, 2006), a mode of vigorous high-amplitude and asymmetrical beating of the tail required for sperm to ascend the oviduct, albeit with low directionality, and penetrate the cumulus oophorus, a mass of granulosa cells enveloping the oocyte, and zona pellucida (Ho et al., 2009; Chang and Suarez, 2012). Deletion of any one of the four genes (*Catsper1*, *Catsper2*, *Catsper3*, or *Catsper4*) encoding its pore-forming subunits (Lin et al., 2021) causes infertility (Jin et al., 2007; Ren et al., 2001; Quill et al., 2003; Qi et al., 2007). Thus, auto- and paracrine cholinergic stimulation of sperm α7 nAChRs, possibly together with stimulation of uterine smooth muscle mAChRs (Agha et al., 2001), may promote sperm migration and transport to the uterotubal junction and oviduct, where capacitation and activation of CatSper stimulate hyperactivated motility, required for further intraoviductal ascend and penetration of the oocyte, as delineated in Figure 2B.

Aside from the generation of choline to support cholinergic signaling, the 5′-ectonucleotidase activity of PAP, which generates adenosine from AMP, may contribute to purinergic signaling in sperm and the female reproductive tract. Adenosine has been shown both to stimulate sperm capacitation (Fraser and Duncan, 1993; Fénichel et al., 1996) and to increase the motility of bovine and human sperm in a dose-dependent fashion (Vijayaraghavan and Hoskins, 1986; Shen et al., 1993). Adenosine receptors (ARs), expressed in sperm (Bellezza and Minelli, 2017), can be categorized into two types of G protein-coupled receptors: A_1_R and A_3_R, which are G_i_-coupled, and A_2a_R and A_2b_R, which are G_s_-coupled. Mice lacking A_1_Rs, encoded by *Adora1*, exhibit normal sperm motility, but take much longer to acquire capacitation (Minelli et al., 2004). This possibly accounts for the ∼40% reduction in average litter size (Minelli et al., 2004). Mice lacking A_3_Rs, encoded by *Adora3*, are fertile and exhibit no obvious developmental abnormalities (Salvatore et al., 2000). Similarly, mice lacking the A_2a_R, encoded by *Adora2a*, are viable and fertile, but exhibit reduced exploratory behavior, among other phenotypes (L et al., 1997; Yaar et al., 2005). Mice engineered to lack A_2b_R, encoded by *Adora2b*, and contain a gene expression reporter (*lacZ*; which encodes β-galactosidase) were found to breed normally (Yang et al., 2006). However, the authors showed that *Adora2b* is expressed in the vasculature and macrophages and *Adora2b*-deficient mice exhibit low-grade inflammation, consistent with an inhibitory action of G_s_-coupled receptor stimulation on proinflammatory signal pathways. Independently, the IMPC have generated *Adora1*, *Adora2a*, and *Adora2b* knockout mouse models and infertility was not observed during phenotype screening. Thus, PAP may potentiate sperm motility by generating adenosine from AMP, although adenosine receptor knockout mouse models have not implicated these receptors in sperm motility or fertility. Further research is needed to clarify the physiological significance of adenosine-induced sperm motility.

## 5 Conclusion

The author proposes that the physiological function of human PAP is to produce choline from PC, which promotes sperm motility via cholinergic signaling. In rodents and other mammals, GPC, rather than PC, is the primary source of choline, which is harvested by uterine diesterases. Across species, the author proposes that choline, in addition to directly stimulating α7 nAChRs, stimulates directional sperm motility via repeated cycles of uptake of choline by the high-affinity choline transporter, generation of ACh from choline via ChAT, release of ACh via an OCTN or similar transporter, auto- and paracrine stimulation of α7 nAChRs, influx of Ca^2+^, and ACh hydrolysis via AChEs. PAP may also generate adenosine from AMP, which may further promote sperm motility via adenosine receptors. These hypotheses are supported by the availability of all the essential molecular components in sperm and seminal plasma, as well as previously published experimental data substantiating the various steps.

## References

[B1] Aaberg R. A. Sauer M. V. Sikka S. Rajfer J. (1989). Effects of extracellular ionized calcium, diltiazem and cAMP on motility of human spermatozoa. J. Urol. 141, 1221–1224. 10.1016/s0022-5347(17)41225-0 2540352

[B2] Agha A. M. Taha R. A. (2001). Sildenafil inhibits agonist-evoked rat uterine contractility: influence of guanylyl cyclase inhibition. Eur. J. Pharmacol. 428, 343–348. 10.1016/s0014-2999(01)01351-6 11689193

[B3] Ahn H. J. Yang H. An B. S. Choi K. C. Jeung E. B. (2011). Expression and regulation of Enpp2 in rat uterus during the estrous cycle. J. veterinary Sci. 12, 379–385. 10.4142/jvs.2011.12.4.379 PMC3232398 22122904

[B4] Alkondon M. Pereira E. F. Cortes W. S. Maelicke A. Albuquerque E. X. (1997). Choline is a selective agonist of alpha7 nicotinic acetylcholine receptors in the rat brain neurons. Eur. J. Neurosci. 9, 2734–2742. 10.1111/j.1460-9568.1997.tb01702.x 9517478

[B5] Anamthathmakula P. Winuthayanon W. (2020). Mechanism of semen liquefaction and its potential for a no*vel non*-hormonal contraception. Biol. reproduction 103, 411–426. 10.1093/biolre/ioaa075 PMC7523691 32529252

[B6] Araujo C. L. Quintero I. B. Kipar A. Herrala A. M. Pulkka A. E. Saarinen L. (2014). Prostatic acid phosphatase is the main acid phosphatase with 5'-ectonucleotidase activity in the male mouse saliva and regulates salivation. Am. J. physiology. Cell physiology 306, C1017–C1027. 10.1152/ajpcell.00062.2014 24717577

[B7] Araujo C. L. Vihko P. T. (2013). Structure of acid phosphatases. Methods Mol. Biol. Clift. N.J.) 1053, 155–166. 10.1007/978-1-62703-562-0_11 23860654

[B8] Arrata W. S. Burt T. Corder S. (1978). The role of phosphate esters in male fertility. Fertil. Steril. 30, 329–333. 10.1016/s0015-0282(16)43521-1 710605

[B9] Austin C. R. (1952). The capacitation of the mammalian sperm. Nature 170, 326. 10.1038/170326a0 12993150

[B10] Bedford J. M. (1963). Changes in the electrophoretic properties of rabbit spermatozoa during passage through the epididymis. Nature 200, 1178–1180. 10.1038/2001178a0 14089901

[B11] Bellezza I. Minelli A. (2017). Adenosine in sperm physiology. Mol. aspects Med. 55, 102–109. 10.1016/j.mam.2016.11.009 27890599

[B12] Bishop M. R. Sastry B. V. Schmidt D. E. Harbison R. D. (1976). Occurrence of choline acetyltransferase and acetylcholine and other quaternary ammonium compounds in mammalian spermatozoa. Biochem. Pharmacol. 25, 1617–1622. 10.1016/0006-2952(76)90473-1 945986

[B13] Borza R. Salgado-Polo F. Moolenaar W. H. Perrakis A. (2022). Structure and function of the ecto-nucleotide pyrophosphatase/phosphodiesterase (ENPP) family: tidying up diversity. J. Biol. Chem. 298, 101526. 10.1016/j.jbc.2021.101526 34958798 PMC8808174

[B14] Bray C. Son J. H. Kumar P. Meizel S. (2005). Mice deficient in CHRNA7, a subunit of the nicotinic acetylcholine receptor, produce sperm with impaired motility. Biol. reproduction 73, 807–814. 10.1095/biolreprod.105.042184 15944242

[B15] Chakraborty J. Nelson L. (1976). Comparative study of cholinesterases distribution in the spermatozoa of some mammalian species. Biol. reproduction 15, 579–585. 10.1093/biolreprod/15.5.579 826286

[B16] Chang H. Suarez S. S. (2012). Unexpected flagellar movement patterns and epithelial binding behavior of mouse sperm in the oviduct. Biol. reproduction 86 (140), 141–148. 10.1095/biolreprod.111.096578 PMC3364923 22337334

[B17] Chang M. C. (1951). Fertilizing capacity of spermatozoa deposited into the fallopian tubes. Nature 168, 697–698. 10.1038/168697b0 14882325

[B18] Chung J. J. Miki K. Kim D. Shim S. H. Shi H. F. Hwang J. Y. (2017). CatSperζ regulates the structural continuity of sperm Ca(2+) signaling domains and is required for normal fertility. Elife 6, e23082. 10.7554/eLife.23082 28226241 PMC5362262

[B19] Dawson R. M. Mann T. White I. G. (1957). Glycerylphosphorylcholine and phosphorylcholine in semen, and their relation to choline. Biochem. J. 65, 627–634. 10.1042/bj0650627 13426075 PMC1199928

[B20] Delbono O. Gopalakrishnan M. Renganathan M. Monteggia L. M. Messi M. L. Sullivan J. P. (1997). Activation of the recombinant human alpha 7 nicotinic acetylcholine receptor significantly raises intracellular free calcium. J. Pharmacol. Exp. Ther. 280, 428–438. 8996225

[B21] Dwivedi C. Long N. J. (1989). Effect of cholinergic agents on human spermatozoa motility. Biochem. Med. Metab. Biol. 42, 66–70. 10.1016/0885-4505(89)90042-x 2775563

[B22] Egbunike G. N. (1980). Changes in acetylcholinesterase activity of mammalian spermatozoa during maturation. Int. J. Androl. 3, 459–468. 10.1111/j.1365-2605.1980.tb00134.x 7440010

[B23] Egbunike G. N. (1982). Effect of chloroquine on the motility and acetylcholinesterase activity of porcine spermatozoa during epididymal maturation. Andrologia 14, 503–508. 10.1111/j.1439-0272.1982.tb02301.x 7165123

[B24] Feng B. Bhattacharyya A. Yanagimachi R. (1988). Ca2+ is essential for the motility of plasma membrane-intact, but not of demembranated, hamster spermatozoa. Andrologia 20, 155–162. 10.1111/j.1439-0272.1988.tb00680.x 3389541

[B25] Fénichel P. Gharib A. Emiliozzi C. Donzeau M. Ménézo Y. (1996). Stimulation of human sperm during capacitation *in vitro* by an adenosine agonist with specificity for A2 receptors. Biol. reproduction 54, 1405–1411. 10.1095/biolreprod54.6.1405 8724371

[B26] Ferguson S. M. Bazalakova M. Savchenko V. Tapia J. C. Wright J. Blakely R. D. (2004). Lethal impairment of cholinergic neurotransmission in hemicholinium-3-sensitive choline transporter knockout mice. Proc. Natl. Acad. Sci. U. S. A. 101, 8762–8767. 10.1073/pnas.0401667101 15173594 PMC423269

[B27] Fraser L. R. Duncan A. E. (1993). Adenosine analogues with specificity for A2 receptors bind to mouse spermatozoa and stimulate adenylate cyclase activity in uncapacitated suspensions. J. Reprod. Fertil. 98, 187–194. 10.1530/jrf.0.0980187 8393927

[B28] Gallazzini M. Ferraris J. D. Burg M. B. (2008). GDPD5 is a glycerophosphocholine phosphodiesterase that osmotically regulates the osmoprotective organic osmolyte GPC. Proc. Natl. Acad. Sci. U. S. A. 105, 11026–11031. 10.1073/pnas.0805496105 18667693 PMC2504799

[B29] Goding J. W. Grobben B. Slegers H. (2003). Physiological and pathophysiological functions of the ecto-nucleotide pyrophosphatase/phosphodiesterase family. Biochimica biophysica acta 1638, 1–19. 10.1016/s0925-4439(03)00058-9 12757929

[B30] Greiner-Tollersrud O. K. (2014). The non-classical N-glycan processing pathway of bovine brain ecto-nucleotide phosphodiesterase/pyrophosphatase 6 (eNPP6) is brain specific and not due to mannose-6-phosphorylation. Neurochem. Res. 39, 2025–2029. 10.1007/s11064-014-1412-1 25142936

[B31] Hinton B. T. Setchell B. P. (1980). Concentrations of glycerophosphocholine, phosphocholine and free inorganic phosphate in the luminal fluid of the rat testis and epididymis. J. Reprod. Fertil. 58, 401–406. 10.1530/jrf.0.0580401 7431273

[B32] Ho K. Wolff C. A. Suarez S. S. (2009). CatSper-null mutant spermatozoa are unable to ascend beyond the oviductal reservoir. Reprod. Fertil. Dev. 21, 345–350. 10.1071/rd08183 19210926

[B33] Hoffmann D. S. Killian G. J. (1981). Isolation of epithelial cells from the corpus epididymidis and analysis for glycerylphosphorylcholine, sialic acid, and protein. J. Exp. zoology 217, 93–102. 10.1002/jez.1402170110 7264580

[B34] Ibáñez C. F. Pelto-Huikko M. Söder O. Ritzèn E. M. Hersh L. B. Hökfelt T. (1991). Expression of choline acetyltransferase mRNA in spermatogenic cells results in an accumulation of the enzyme in the postacrosomal region of mature spermatozoa. Proc. Natl. Acad. Sci. U. S. A. 88, 3676–3680. 10.1073/pnas.88.9.3676 2023918 PMC51515

[B35] Ignotz G. G. Suarez S. S. (2005). Calcium/calmodulin and calmodulin kinase II stimulate hyperactivation in demembranated bovine sperm. Biol. reproduction 73, 519–526. 10.1095/biolreprod.105.040733 15878888

[B36] Jakob C. G. Lewinski K. Kuciel R. Ostrowski W. Lebioda L. (2000). Crystal structure of human prostatic acid phosphatase. Prostate 42, 211–218. 10.1002/(sici)1097-0045(20000215)42:3<211::aid-pros7>3.0.co;2-u 10639192

[B37] Jin J. Jin N. Zheng H. Ro S. Tafolla D. Sanders K. M. (2007). Catsper3 and Catsper4 are essential for sperm hyperactivated motility and male fertility in the mouse. Biol. reproduction 77, 37–44. 10.1095/biolreprod.107.060186 17344468

[B38] Juyena N. S. Stelletta C. (2012). Seminal plasma: an essential attribute to spermatozoa. J. Androl. 33, 536–551. 10.2164/jandrol.110.012583 22016346

[B39] Kato Y. Kubo Y. Iwata D. Kato S. Sudo T. Sugiura T. (2010). Gene knockout and metabolome analysis of carnitine/organic cation transporter OCTN1. Pharm. Res. 27, 832–840. 10.1007/s11095-010-0076-z 20224991

[B40] Kawano N. Araki N. Yoshida K. Hibino T. Ohnami N. Makino M. (2014). Seminal vesicle protein SVS2 is required for sperm survival in the uterus. Proc. Natl. Acad. Sci. U. S. A. 111, 4145–4150. 10.1073/pnas.1320715111 24591616 PMC3964112

[B41] Kirichok Y. Navarro B. Clapham D. E. (2006). Whole-cell patch-clamp measurements of spermatozoa reveal an alkaline-activated Ca2+ channel. Nature 439, 737–740. 10.1038/nature04417 16467839

[B42] Kobayashi D. Tamai I. Sai Y. Yoshida K. Wakayama T. Kido Y. (2007). Transport of carnitine and acetylcarnitine by carnitine/organic cation transporter (OCTN) 2 and OCTN3 into epididymal spermatozoa. Reprod. Camb. Engl. 134, 651–658. 10.1530/REP-06-0173 17965255

[B43] Kumar P. Meizel S. (2005). Nicotinic acetylcholine receptor subunits and associated proteins in human sperm. J. Biol. Chem. 280, 25928–25935. 10.1074/jbc.M502435200 15894803

[B44] Kutscher W. Wolbergs H. (1935). Prostataphosphatase. Z. für Physiol. Chem. 236, 237–240. 10.1515/bchm2.1935.236.4-6.237

[B45] Lan R. Xin M. Hao Z. You S. Xu Y. Wu J. (2020). Biological functions and large-scale profiling of protein glycosylation in human semen. J. proteome Res. 19, 3877–3889. 10.1021/acs.jproteome.9b00795 32875803

[B46] Lang Q. Zhang H. Li J. Yin H. Zhang Y. Tang W. (2008). Cloning and characterization of a human GDPD domain-containing protein GDPD5. Mol. Biol. Rep. 35, 351–359. 10.1007/s11033-007-9093-3 17578682

[B47] Ledent C. Vaugeois J. M. Schiffmann S. N. Pedrazzini T. El Yacoubi M. Vanderhaeghen J. J. (1997). Aggressiveness, hypoalgesia and high blood pressure in mice lacking the adenosine A2a receptor. Nature 388, 674–678. 10.1038/41771 9262401

[B48] Lilja H. Laurell C. B. (1984). Liquefaction of coagulated human semen. Scand. J. Clin. laboratory investigation 44, 447–452. 10.3109/00365518409083836 6385215

[B49] Lilja H. Laurell C. B. (1985). The predominant protein in human seminal coagulate. Scand. J. Clin. laboratory investigation 45, 635–641. 10.3109/00365518509155271 2866578

[B50] Lilja H. Oldbring J. Rannevik G. Laurell C. B. (1987). Seminal vesicle-secreted proteins and their reactions during gelation and liquefaction of human semen. J. Clin. investigation 80, 281–285. 10.1172/JCI113070 PMC442235 3611349

[B51] Lin S. Ke M. Zhang Y. Yan Z. Wu J. (2021). Structure of a mammalian sperm cation channel complex. Nature 595, 746–750. 10.1038/s41586-021-03742-6 34225353

[B52] Lips K. S. Volk C. Schmitt B. M. Pfeil U. Arndt P. Miska D. (2005). Polyspecific cation transporters mediate luminal release of acetylcholine from bronchial epithelium. Am. J. Respir. Cell Mol. Biol. 33, 79–88. 10.1165/rcmb.2004-0363OC 15817714

[B53] Lundquist F. (1946). Function of prostatic phosphatase. Nature 158, 710–711. 10.1038/158710a0

[B54] Lundquist F. (1953). Glycerophosphoryl choline as a precursor of free choline in mammalian semen. Nature 172, 587–588. 10.1038/172587a0 13099269

[B55] Makino Y. Hiradate Y. Umezu K. Hara K. Tanemura K. (2021). Expression and possible role of nicotinic acetylcholine receptor ε subunit (AChRe) in mouse sperm. Biol. (Basel) 10, 46. 10.3390/biology10010046 PMC7826850 33440720

[B56] Malm J. Jonsson M. Frohm B. Linse S. (2007). Structural properties of semenogelin I. FEBS J. 274, 4503–4510. 10.1111/j.1742-4658.2007.05979.x 17680810

[B57] McGrady A. V. Nelson L. (1976). Cholinergic effects on bull and chimpanzee sperm motility. Biol. reproduction 15, 248–253. 10.1095/biolreprod15.2.248 963150

[B58] McTigue J. J. Van Etten R. L. (1978). An essential active-site histidine residue in human prostatic acid phosphatase. Ethoxyformylation by diethyl pyrocarbonate and phosphorylation by a substrate. Biochimica biophysica acta 523, 407–421. 10.1016/0005-2744(78)90043-8 656435

[B59] Mike A. Castro N. G. Albuquerque E. X. (2000). Choline and acetylcholine have similar kinetic properties of activation and desensitization on the alpha7 nicotinic receptors in rat hippocampal neurons. Brain Res. 882, 155–168. 10.1016/s0006-8993(00)02863-8 11056195

[B60] Minelli A. Liguori L. Bellazza I. Mannucci R. Johansson B. Fredholm B. B. (2004). Involvement of A1 adenosine receptors in the acquisition of fertilizing capacity. J. Androl. 25, 286–292. 10.1002/j.1939-4640.2004.tb02789.x 14760015

[B61] Misgeld T. Burgess R. W. Lewis R. M. Cunningham J. M. Lichtman J. W. Sanes J. R. (2002). Roles of neurotransmitter in synapse formation: development of neuromuscular junctions lacking choline acetyltransferase. Neuron 36, 635–648. 10.1016/s0896-6273(02)01020-6 12441053

[B62] Mitra J. Chowdhury M. (1994). Ca2+ dependent activation of rat uterine glycerylphosphorylcholine diesterase: presence of a positive modulator protein in uterine secretion. Mol. Cell. Biochem. 139, 101–108. 10.1007/BF01081732 7862100

[B63] Morita J. Kano K. Kato K. Takita H. Sakagami H. Yamamoto Y. (2016). Structure and biological function of ENPP6, a choline-specific glycerophosphodiester-phosphodiesterase. Sci. Rep. 6, 20995. 10.1038/srep20995 26888014 PMC4757880

[B64] Nelson L. (1964). Acetylcholinesterase in bull spermatozoa. J. Reprod. Fertil. 7, 65–71. 10.1530/jrf.0.0070065 14125186

[B65] Nelson L. (1972). Neurochemical control of Arbacia sperm motility. Exp. Cell Res. 74, 269–274. 10.1016/0014-4827(72)90504-6 4342185

[B66] Nelson L. (1976). alpha-bungarotoxin binding by cell membranes. Blockage of sperm cell motility. Exp. Cell Res. 101, 221–224. 10.1016/0014-4827(76)90371-2 986947

[B67] Orr-Urtreger A. Göldner F. M. Saeki M. Lorenzo I. Goldberg L. De Biasi M. (1997). Mice deficient in the alpha7 neuronal nicotinic acetylcholine receptor lack alpha-bungarotoxin binding sites and hippocampal fast nicotinic currents. J. Neurosci. 17, 9165–9171. 10.1523/JNEUROSCI.17-23-09165.1997 9364063 PMC6573618

[B68] Papke R. L. Bencherif M. Lippiello P. (1996). An evaluation of neuronal nicotinic acetylcholine receptor activation by quaternary nitrogen compounds indicates that choline is selective for the alpha 7 subtype. Neurosci. Lett. 213, 201–204. 10.1016/0304-3940(96)12889-5 8873149

[B69] Papke R. L. Kem W. R. Soti F. López-Hernández G. Y. Horenstein N. A. (2009). Activation and desensitization of nicotinic alpha7-type acetylcholine receptors by benzylidene anabaseines and nicotine. J. Pharmacol. Exp. Ther. 329, 791–807. 10.1124/jpet.108.150151 19223664 PMC2672872

[B70] Piibor J. Dissanayake K. Midekessa G. Andronowska A. Kavak A. Waldmann A. (2023). Characterization of bovine uterine fluid extracellular vesicles proteomic profiles at follicular and luteal phases of the oestrous cycle. Veterinary Res. Commun. 47, 885–900. 10.1007/s11259-022-10052-3 PMC10209254 36547796

[B71] Pochini L. Galluccio M. Scalise M. Console L. Indiveri C. (2019). OCTN: a small transporter subfamily with great relevance to human pathophysiology, drug discovery, and diagnostics. SLAS Discov. Adv. life Sci. R D 24, 89–110. 10.1177/2472555218812821 30523710

[B72] Pochini L. Scalise M. Galluccio M. Pani G. Siminovitch K. A. Indiveri C. (2012). The human OCTN1 (SLC22A4) reconstituted in liposomes catalyzes acetylcholine transport which is defective in the mutant L503F associated to the Crohn's disease. Biochimica biophysica acta 1818, 559–565. 10.1016/j.bbamem.2011.12.014 22206629

[B73] Puga Molina L. C. Luque G. M. Balestrini P. A. Marín-Briggiler C. I. Romarowski A. Buffone M. G. (2018). Molecular basis of human sperm capacitation. Front. Cell Dev. Biol. 6, 72. 10.3389/fcell.2018.00072 30105226 PMC6078053

[B74] Qi H. Moran M. M. Navarro B. Chong J. A. Krapivinsky G. Krapivinsky L. (2007). All four CatSper ion channel proteins are required for male fertility and sperm cell hyperactivated motility. Proc. Natl. Acad. Sci. U. S. A. 104, 1219–1223. 10.1073/pnas.0610286104 17227845 PMC1770895

[B75] Quill T. A. Sugden S. A. Rossi K. L. Doolittle L. K. Hammer R. E. Garbers D. L. (2003). Hyperactivated sperm motility driven by CatSper2 is required for fertilization. Proc. Natl. Acad. Sci. U. S. A. 100, 14869–14874. 10.1073/pnas.2136654100 14657366 PMC299835

[B76] Quintero I. B. Araujo C. L. Pulkka A. E. Wirkkala R. S. Herrala A. M. Eskelinen E. L. (2007). Prostatic acid phosphatase is not a prostate specific target. Cancer Res. 67, 6549–6554. 10.1158/0008-5472.CAN-07-1651 17638863

[B77] Quintero I. B. Herrala A. M. Araujo C. L. Pulkka A. E. Hautaniemi S. Ovaska K. (2013). Transmembrane prostatic acid phosphatase (TMPAP) interacts with snapin and deficient mice develop prostate adenocarcinoma. PloS one 8, e73072. 10.1371/journal.pone.0073072 24039861 PMC3769315

[B78] Ren D. Navarro B. Perez G. Jackson A. C. Hsu S. Shi Q. (2001). A sperm ion channel required for sperm motility and male fertility. Nature 413, 603–609. 10.1038/35098027 11595941 PMC8462998

[B79] Rigden D. J. (2008). The histidine phosphatase superfamily: structure and function. Biochem. J. 409, 333–348. 10.1042/BJ20071097 18092946

[B80] Rodriguez M. Choi J. Park S. Sockanathan S. (2012). Gde2 regulates cortical neuronal identity by controlling the timing of cortical progenitor differentiation. Dev. Camb. Engl. 139, 3870–3879. 10.1242/dev.081083 22951639

[B81] Sabharwal P. Lee C. Park S. Rao M. Sockanathan S. (2011). GDE2 regulates subtype-specific motor neuron generation through inhibition of Notch signaling. Neuron 71, 1058–1070. 10.1016/j.neuron.2011.07.028 21943603 PMC3183458

[B82] Sadate-Ngatchou P. I. Payne C. J. Dearth A. T. Braun R. E. (2008). Cre recombinase activity specific to postnatal, premeiotic male germ cells in transgenic mice. Genes. (New York, N.Y. 46, 738–742. 10.1002/dvg.20437 PMC2837914 18850594

[B83] Saini M. S. Van Etten R. L. (1978). A homogeneous isoenzyme of human liver acid phosphatase. Archives Biochem. biophysics 191, 613–624. 10.1016/0003-9861(78)90399-5 33598

[B84] Saini M. S. Van Etten R. L. (1981). A clinical assay for prostatic acid phosphatase using choline phosphate as a substrate: comparison with thymolphthalein phosphate. Prostate 2, 359–368. 10.1002/pros.2990020404 7329872

[B85] Salvatore C. A. Tilley S. L. Latour A. M. Fletcher D. S. Koller B. H. Jacobson M. A. (2000). Disruption of the A(3) adenosine receptor gene in mice and its effect on stimulated inflammatory cells. J. Biol. Chem. 275, 4429–4434. 10.1074/jbc.275.6.4429 10660615

[B86] Sastry B. V. Janson V. E. Chaturvedi A. K. (1981). Inhibition of human sperm motility by inhibitors of choline acetyltransferase. J. Pharmacol. Exp. Ther. 216, 378–384. 7463354

[B87] Schirmer S. U. Eckhardt I. Lau H. Klein J. DeGraaf Y. C. Lips K. S. (2011). The cholinergic system in rat testis is of non-neuronal origin. Reprod. Camb. Engl. 142, 157–166. 10.1530/REP-10-0302 21482687

[B88] Scott T. W. Wales R. G. Wallace J. C. White I. G. (1963). Composition of ram epididymal and testicular fluid and the biosynthesis of glycerylphosphorylcholine by the rabbit epididymis. J. Reprod. Fertil. 6, 49–59. 10.1530/jrf.0.0060049 14064213

[B89] Seligman A. M. Sternberger N. J. Paul B. D. Friedman A. E. Shannon W. A. Jr. Wasserkrug H. L. (1975). Design of spindle poisons activated specifically by prostatic acid phosphatase (PAP) and new methods for PAP cytochemistry. Cancer Chemother. Rep. 59, 233–242. 805658

[B90] Seo H. Choi Y. Shim J. Kim M. Ka H. (2012). Analysis of the lysophosphatidic acid-generating enzyme ENPP2 in the uterus during pregnancy in pigs. Biol. reproduction 87, 77. 10.1095/biolreprod.112.099564 22914316

[B91] Serrano J. A. Wasserkrug H. L. Serrano A. A. Paul B. D. Seligman A. M. (1977). The histochemical demonstration of human prostatic acid phosphatase with phosphorylcholine. Investig. Urol. 15, 123–136. 71283

[B92] Shen M. R. Linden J. Chen S. S. Wu S. N. (1993). Identification of adenosine receptors in human spermatozoa. Clin. Exp. Pharmacol. physiology 20, 527–534. 10.1111/j.1440-1681.1993.tb01736.x 8403534

[B93] Skok M. (2022). Universal nature of cholinergic regulation demonstrated with nicotinic acetylcholine receptors. BBA Adv. 2, 100061. 10.1016/j.bbadva.2022.100061 37082580 PMC10074969

[B94] Sliwa L. (1995). Chemotaction of mouse spermatozoa induced by certain hormones. Archives Androl. 35, 105–110. 10.3109/01485019508987860 8579470

[B95] Stewart T. A. Forrester I. T. (1978). Acetylcholinesterase and choline acetyltransferase in ram spermatozoa. Biol. reproduction 19, 271–279. 10.1095/biolreprod19.2.271 719088

[B96] Street S. E. Kramer N. J. Walsh P. L. Taylor-Blake B. Yadav M. C. King I. F. (2013). Tissue-nonspecific alkaline phosphatase acts redundantly with PAP and NT5E to generate adenosine in the dorsal spinal cord. J. Neurosci. 33, 11314–11322. 10.1523/JNEUROSCI.0133-13.2013 23825434 PMC3718384

[B97] Suarez S. S. (2008). Regulation of sperm storage and movement in the mammalian oviduct. Int. J. Dev. Biol. 52, 455–462. 10.1387/ijdb.072527ss 18649258

[B98] Suarez S. S. (2016). Mammalian sperm interactions with the female reproductive tract. Cell tissue Res. 363, 185–194. 10.1007/s00441-015-2244-2 26183721 PMC4703433

[B99] Tanaka M. Kishi Y. Takanezawa Y. Kakehi Y. Aoki J. Arai H. (2004). Prostatic acid phosphatase degrades lysophosphatidic acid in seminal plasma. FEBS Lett. 571, 197–204. 10.1016/j.febslet.2004.06.083 15280042

[B100] Tecle E. Gagneux P. (2015). Sugar-coated sperm: unraveling the functions of the mammalian sperm glycocalyx. Mol. reproduction Dev. 82, 635–650. 10.1002/mrd.22500 PMC4744710 26061344

[B101] Torres-Flores V. Picazo-Juárez G. Hernández-Rueda Y. Darszon A. González-Martínez M. T. (2011). Sodium influx induced by external calcium chelation decreases human sperm motility. Hum. Reprod. 26, 2626–2635. 10.1093/humrep/der237 21810864 PMC3174032

[B102] Turner R. M. (2006). Moving to the beat: a review of mammalian sperm motility regulation. Reprod. Fertil. Dev. 18, 25–38. 10.1071/rd05120 16478600

[B103] van Meeteren L. A. Ruurs P. Stortelers C. Bouwman P. van Rooijen M. A. Pradère J. P. (2006). Autotaxin, a secreted lysophospholipase D, is essential for blood vessel formation during development. Mol. Cell. Biol. 26, 5015–5022. 10.1128/MCB.02419-05 16782887 PMC1489177

[B104] Vijayaraghavan S. Hoskins D. D. (1986). Regulation of bovine sperm motility and cyclic adenosine 3',5'-monophosphate by adenosine and its analogues. Biol. reproduction 34, 468–477. 10.1095/biolreprod34.3.468 2421788

[B105] Wallace J. C. Wales R. G. White I. G. (1966). The respiration of the rabbit epididymis and its synthesis of glycerylphosphorylcholine. Aust. J. Biol. Sci. 19, 849–856. 10.1071/bi9660849 6008062

[B106] Wang H. McGoldrick L. L. Chung J. J. (2021). Sperm ion channels and transporters in male fertility and infertility. Nat. Rev. Urol. 18, 46–66. 10.1038/s41585-020-00390-9 33214707 PMC7852504

[B107] Wang S. Larina I. V. (2023). Dynamics of gametes and embryos in the oviduct: what can *in vivo* imaging reveal? Reprod. Camb. Engl. 165, R25–r37. 10.1530/REP-22-0250 PMC9827618 36318634

[B108] Wessler I. K. Kirkpatrick C. J. (2017). Non-neuronal acetylcholine involved in reproduction in mammals and honeybees. J. Neurochem. 142 (2), 144–150. 10.1111/jnc.13953 28072454

[B109] White I. G. Wallace J. C. (1961). Breakdown of seminal glyceryl-phosphorylcholine by secretions of the female reproductive tract. Nature 189, 843–844. 10.1038/189843a0 13784829

[B110] Xuan W. Lamhonwah A. M. Librach C. Jarvi K. Tein I. (2003). Characterization of organic cation/carnitine transporter family in human sperm. Biochem. biophysical Res. Commun. 306, 121–128. 10.1016/s0006-291x(03)00930-6 12788076

[B111] Yaar R. Jones M. R. Chen J. F. Ravid K. (2005). Animal models for the study of adenosine receptor function. J. Cell. physiology 202, 9–20. 10.1002/jcp.20138 15389588

[B112] Yanagimachi R. (2022). Mysteries and unsolved problems of mammalian fertilization and related topics. Biol. reproduction 106, 644–675. 10.1093/biolre/ioac037 PMC9040664 35292804

[B113] Yang D. Zhang Y. Nguyen H. G. Koupenova M. Chauhan A. K. Makitalo M. (2006). The A2B adenosine receptor protects against inflammation and excessive vascular adhesion. J. Clin. investigation 116, 1913–1923. 10.1172/JCI27933 PMC1483170 16823489

[B114] Yeung C. H. Cooper T. G. Oberpenning F. Schulze H. Nieschlag E. (1993). Changes in movement characteristics of human spermatozoa along the length of the epididymis. Biol. reproduction 49, 274–280. 10.1095/biolreprod49.2.274 8373950

[B115] Zylka M. J. Sowa N. A. Taylor-Blake B. Twomey M. A. Herrala A. Voikar V. (2008). Prostatic acid phosphatase is an ectonucleotidase and suppresses pain by generating adenosine. Neuron 60, 111–122. 10.1016/j.neuron.2008.08.024 18940592 PMC2629077

